# Correction: “It’s a mother’s choice”: Exploring personal experiences, community support, cultural influences, and breastfeeding alternatives in Florence, Italy

**DOI:** 10.1371/journal.pgph.0005114

**Published:** 2025-08-21

**Authors:** Megan Morley, Anjali Natarajan, Nicole A. Stepp, Paige Kertes, Isabella M. Ford, Andrea L. DeMaria

Paige Kertes should be included in the author byline. Paige Kertes should be listed as the fourth author, and affiliated with: the Department of Chemistry, College of Science, Purdue University, West Lafayette, Indiana, United States of America. The contributions of this author are as follows: Data Curation, Formal Analysis, Investigation and Writing – Original Draft Preparation.

Isabella M. Ford should be included in the author byline. Isabella M. Ford should be listed as the fifth author, and affiliated with: the Department of Biological Sciences, College of Science, Purdue University, West Lafayette, Indiana, United States of America and Department of Political Science, College of Liberal Arts, Purdue University, West Lafayette, Indiana, United States of America. The contribution of this author are as follows: Data Curation, Formal Analysis, Investigation and Writing – Original Draft Preparation.

The correct citation is: Morley M, Natarajan A, Stepp NA, Kertes P, Ford IM, DeMaria AL (2025) “It’s a mother’s choice”: Exploring personal experiences, community support, cultural influences, and breastfeeding alternatives in Florence, Italy. PLOS Glob Public Health 5(2): e0004282. https://doi.org/10.1371/journal.pgph.0004282.
