# Supplementary material for: “It’s a mother’s choice”: Exploring personal experiences, community support, cultural influences, and breastfeeding alternatives in Florence, Italy
Source: PLOS Glob Public Health. 2025 Feb 28;5(2):e0004282. doi: 10.1371/journal.pgph.0004282 (PMC11870355; doi:10.1371/journal.pgph.0004282)
Supplement: Attachment — Submitted filename: Reviewer feedback for PLOS Global Health (Final).docx [file pgph.0004282.s002.docx]

# Reviewer feedback for NR 2024

**Recommendation**

Accept with minor amendments.

**Comments for Editor**

Thank you for the opportunity to review this manuscript entitled *“It’s a mother’s choice”: Exploring Personal Experiences, Community Support, Cultural Influences, and Breastfeeding Alternatives in Florence, Italy.* The aim of this study was to explore women’s perceptions and behaviors surrounding breastfeeding in and around Florence, Italy to fill a gap in breastfeeding research.

The abstract is clear and concise. The introduction sets societal aspects of breastfeeding in context but I think the use of up to date references would enhance the background. Currently, the cited references date from 2004 to 2016. Similarly in terms of Europe and Italy a reference (e.g. Cattaneo et al., 2000 and Yngve et al. 2001) are used to describe breastfeeding data collection challenges. The World Breastfeeding Trends initiative <https://www.worldbreastfeedingtrends.org/> and latest reports [Publications (worldbreastfeedingtrends.org)](https://www.worldbreastfeedingtrends.org/resources/publications) would have been potentially useful citations and would also add up to date opportunities to discuss country related issues with collecting breastfeeding data. I acknowledge that the study was driven by a study broad programme which was novel. However, from a research perspective a better rationale is needed for choosing Florence e.g. is there anything unique about the city in terms of breastfeeding? Have there been recent studies whose results suggested more depth might be required?

In terms of materials and methods the researchers could have provided more information on the qualitative methodology used for example did you intend it to be descriptive or interpretive. Good detail is provided regarding the methods employed however the semi structured protocol referred to has more closed than open probes. Excellent detail is provided regarding the research team however more detail could have been provided regarding the potential impact of using 24 individual interviewers. If not here, then in the limitations section. It is unclear if the four UG students involved in data analysis were from the original 24 students who undertook the interviews? Therefore, please provide greater clarity. Lines 129 to 133 need greater clarity to describe the order of thematic development. It would appear that subthemes came after theme development rather than the other way around see <https://www.bmj.com/content/381/bmj-2022-074256>

Four themes are listed in the results and numbered but the subsequent headings in this section (and table 3) differ. E.g. **“Happy Mom, Happy Baby”: Personal Experiences, Opinions, and Attitudes** is the theme heading it does not correspond exactly with 1 as listed. Using a narrative phrase is fine but it needs to be used consistently. You could consider it at the end e.g. **Personal Experiences, Opinions, and Attitudes** “Happy Mom, Happy Baby”. Line 147 should be followed by an introductory sentence signposting how you will be presenting findings e.g. then the subthemes; duration, breastmilk production … etc. This approach is needed for each of the four themes.

In terms of the discussion there is a reliance to the dated reference (Aksu et al. 2011). Current and broader evidence needs to be incorporated into the discussion. Similarly, the paragraph contained within lines 466 to 476 relies on just one citation which needs to be amended.

In the Strengths, Limitations, and Future Research, the strengths are somewhat overstated regarding recruiting and sampling strategy. You've indicated snowball sampling was used but were other strategies considered bearing in mind the inherited inherent biases with snowball sampling. The impact of 24 separate interviewers also needs greater acknowledgement. For example, where there are small numbers of interviewers there is learning and development of confidence which can result in more depth. The probing questions in table 1 are mainly closed rather than open. In terms of transferability, it would be important to know if participants were typical or untypical of breastfeeding women on Florence. It would appear from your result that the participants in this study were older, educated parents, and ‘comfortable’ in terms of socio-economic status so this needs discussion. All sub-theme labels need to be meaningful therefore consider revising ‘circumstantial.

In summary the recommended amendments relate to:

- Including up to date contextual literature
- Providing a methodological rationale for the choice of the study site
- Some amendment of theme and sub-theme headings in results
- Strengthen the discussion by including up to date citations
- Avoid overstating the strengths of the study and properly acknowledge methodological limitations
