# Supplementary material for: “It’s a mother’s choice”: Exploring personal experiences, community support, cultural influences, and breastfeeding alternatives in Florence, Italy
Source: PLOS Glob Public Health. 2025 Feb 28;5(2):e0004282. doi: 10.1371/journal.pgph.0004282 (PMC11870355; doi:10.1371/journal.pgph.0004282)
Supplement: Attachment — Submitted filename: Response to Reviewers 11.13.24.docx [file pgph.0004282.s004.docx]

Reviewer #1

Thank you for the opportunity to review this manuscript entitled *“It’s a mother’s choice”: Exploring Personal Experiences, Community Support, Cultural Influences, and Breastfeeding Alternatives in Florence, Italy.* The aim of this study was to explore women’s perceptions and behaviors surrounding breastfeeding in and around Florence, Italy to fill a gap in breastfeeding research.

Response>> Thank you for your thoughtful review. We appreciate your positive, constructive feedback and are grateful for recognizing our research’s significance in this field.

The abstract is clear and concise. The introduction sets societal aspects of breastfeeding in context but I think the use of up to date references would enhance the background. Currently, the cited references date from 2004 to 2016. Similarly in terms of Europe and Italy a reference (e.g. Cattaneo et al., 2000 and Yngve et al. 2001) are used to describe breastfeeding data collection challenges. The World Breastfeeding Trends initiative <https://www.worldbreastfeedingtrends.org/> and latest reports [Publications (worldbreastfeedingtrends.org)](https://www.worldbreastfeedingtrends.org/resources/publications) would have been potentially useful citations and would also add up to date opportunities to discuss country related issues with collecting breastfeeding data. I acknowledge that the study was driven by a study broad programme which was novel. However, from a research perspective a better rationale is needed for choosing Florence e.g. is there anything unique about the city in terms of breastfeeding? Have there been recent studies whose results suggested more depth might be required?

Response>> We agree that recent literature could add to the depth of our research. Consequently, we have added several more recent sources regarding European, Italian, and Tuscan breastfeeding rates. The regional data for Tuscany has also been added to our rationale for choosing Florence (the capital city of Tuscany) to provide insight into culture, community influence, and public opinion as a possible explanation for regional variabilities regarding breastfeeding.

In terms of materials and methods the researchers could have provided more information on the qualitative methodology used for example did you intend it to be descriptive or interpretive. Good detail is provided regarding the methods employed however the semi structured protocol referred to has more closed than open probes. Excellent detail is provided regarding the research team however more detail could have been provided regarding the potential impact of using 24 individual interviewers. If not here, then in the limitations section. It is unclear if the four UG students involved in data analysis were from the original 24 students who undertook the interviews? Therefore, please provide greater clarity. Lines 129 to 133 need greater clarity to describe the order of thematic development. It would appear that subthemes came after theme development rather than the other way around see <https://www.bmj.com/content/381/bmj-2022-074256>

Response>>This comment has been addressed. We have clarified the qualitative methodology follows a descriptive approach. Additional details now explain that while some semi-structured protocol questions were closed, they served as prompts to foster deeper discussions. We added a note in the limitations section regarding the potential impact of using 24 interviewers and confirmed that the four undergraduate students analyzing the data were among the original interviewers. The sections have been revised to clarify that themes were developed before subthemes, following the thematic analysis process outlined in the BMJ article provided.

Four themes are listed in the results and numbered but the subsequent headings in this section (and table 3) differ. E.g. **“Happy Mom, Happy Baby”: Personal Experiences, Opinions, and Attitudes** is the theme heading it does not correspond exactly with 1 as listed. Using a narrative phrase is fine but it needs to be used consistently. You could consider it at the end e.g. **Personal Experiences, Opinions, and Attitudes** “Happy Mom, Happy Baby”. Line 147 should be followed by an introductory sentence signposting how you will be presenting findings e.g. then the subthemes; duration, breastmilk production … etc. This approach is needed for each of the four themes.

Response>> Thank you for bringing this to our attention. We have addressed this comment by clarifying theme headings and appropriately introducing subthemes in the results.

In terms of the discussion there is a reliance to the dated reference (Aksu et al. 2011). Current and broader evidence needs to be incorporated into the discussion. Similarly, the paragraph contained within lines 466 to 476 relies on just one citation which needs to be amended.

Response>> Thank you for pointing out this oversight in our discussion. The Aksu-dated reference has been removed and replaced with more recent evidence from similar studies. Additionally, we have added more evidence to the later-noted paragraph to bolster its impact in our discussion.

In the Strengths, Limitations, and Future Research, the strengths are somewhat overstated regarding recruiting and sampling strategy. You've indicated snowball sampling was used but were other strategies considered bearing in mind the inherited inherent biases with snowball sampling. The impact of 24 separate interviewers also needs greater acknowledgement. For example, where there are small numbers of interviewers there is learning and development of confidence which can result in more depth. The probing questions in table 1 are mainly closed rather than open. In terms of transferability, it would be important to know if participants were typical or untypical of breastfeeding women on Florence. It would appear from your result that the participants in this study were older, educated parents, and ‘comfortable’ in terms of socio-economic status so this needs discussion. All sub-theme labels need to be meaningful therefore consider revising ‘circumstantial.

Response>> Thank you for your insightful feedback. Regarding our sampling strategy, we acknowledge the inherent biases associated with snowball sampling. Although other strategies were considered, time constraints due to our limited presence in Italy necessitated snowball sampling as the most feasible approach for timely data collection. We have revised the manuscript to address this limitation in the discussion section. We have also expanded our discussion on the impact of involving 24 separate interviewers; having a smaller group of interviewers might have developed more consistent probing techniques and deeper insights over time. Regarding transferability, we agree that our participants primarily represented older, educated parents with relatively high socioeconomic status, typical of breastfeeding women. We have expanded our discussion to contextualize this demographic information and its relevance to our findings’ transferability. Lastly, we reviewed all sub-theme labels to ensure clarity and resonance with the study’s aims, revising “circumstantial” to “individualized experiences” as that emphasizes that decisions are made according to each mother’s specific experience.

In summary the recommended amendments relate to:

- Including up to date contextual literature
- Providing a methodological rationale for the choice of the study site
- Some amendment of theme and sub-theme headings in results
- Strengthen the discussion by including up to date citations
- Avoid overstating the strengths of the study and properly acknowledge methodological limitations

Response>> Thank you for your feedback. In response to your comments, the manuscript has been updated to include recent contextual literature that enhances the study's relevance. A detailed rationale for the choice of study site has been added to the methods section to clarify its significance. The theme and sub-theme headings in the results have been revised for improved clarity. Additionally, the discussion has been strengthened by incorporating up-to-date citations, ensuring a more comprehensive analysis. Finally, the language has been adjusted to accurately reflect the study's strengths while properly acknowledging its methodological limitations.

Reviewer #2

The identified themes are unclear and not the same as listed in the main text. Consider adding a line on the methods. The study aim is stated more than once in the abstract.

Response>> Thank you for reviewing our paper. We value your positive and constructive feedback and appreciate your support in increasing awareness and research on this topic. Thank you for further bringing this point of confusion to our attention. Theme headings have been clarified throughout the abstract and main text. The study aim has been clarified as well and is now only stated once in the abstract

Authors should consider reviewing and including recent literature in this section.

Response>> We have reviewed and included recent applicable literature in the introduction, most notably data from the World Breastfeeding Trends Initiative reports.

Authors should consider adding details on trust worthiness of the study.

Response>> Thank you for your feedback. We have incorporated additional details on trustworthiness within the strengths, limitations, and future research sections of our paper. This includes specific explanations addressing credibility and transferability to further enhance the reliability of our research findings. We have highlighted the expertise of our principal investigator, a decorated global health and women’s health expert with extensive research experience in Italy, to further emphasize the study’s credibility and the depth of cultural insight guiding the research process.

The stated themes are not the same as the themes which were stated in the abstract. Please be consistent. Authors should consider adding the definition for each theme.

Response>> Theme headings and names have been changed to be consistent and the same throughout the abstract and main text.
